# Exhaled air dispersion during bag-mask ventilation and sputum suctioning - Implications for infection control

**DOI:** 10.1038/s41598-017-18614-1

**Published:** 2018-01-09

**Authors:** Matthew T. V. Chan, Benny K. Chow, Thomas Lo, Fanny W. Ko, Susanna S. Ng, Tony Gin, David S. Hui

**Affiliations:** 1 Department of Anaesthesia and Intensive Care, The Chinese University of Hong Kong, Hong Kong, China; 2 Stanley Ho Center for Emerging Infectious Diseases, The Chinese University of Hong Kong, Hong Kong, China; 3 Department of Medicine and Therapeutics, The Chinese University of Hong Kong, Hong Kong, China; 4 Li Ka Shing Institute of Health Sciences, The Chinese University of Hong Kong, Hong Kong, China

## Abstract

Mask ventilation and coughing during oro-tracheal suctioning produce aerosols that enhance nosocomial transmission of respiratory infections. We examined the extent of exhaled air dispersion from a human-patient-simulator during mask ventilation by different groups of healthcare workers and coughing bouts. The simulator was programmed to mimic varying severity of lung injury. Exhaled airflow was marked with tiny smoke particles, and highlighted by laser light-sheet. We determined the normalized exhaled air concentration in the leakage jet plume from the light scattered by smoke particles. Smoke concentration ≥20% was considered as significant exposure. Exhaled air leaked from mask-face interface in the transverse plane was most severe (267 ± 44 mm) with Ambu silicone resuscitator performed by nurses. Dispersion was however similar among anesthesiologists/intensivists, respiratory physicians and medical students using Ambu or Laerdal silicone resuscitator, *p* = 0.974. The largest dispersion was 860 ± 93 mm during normal coughing effort without tracheal intubation and decreased with worsening coughing efforts. Oro-tracheal suctioning reduced dispersion significantly, *p* < 0.001, and was more effective when applied continuously. Skills to ensure good fit during mask ventilation are important in preventing air leakage through the mask-face interface. Continuous oro-tracheal suctioning minimized exhaled air dispersion during coughing bouts when performing aerosol-generating procedures.

## Introduction

Respiratory failure is a serious complication of emerging infectious respiratory diseases such as severe acute respiratory syndrome (SARS)^1,2^, avian influenza^3^, influenza A(H1N1)2009 infection^4^ and the Middle East Respiratory Syndrome^5^. Supplemental oxygen, non-invasive ventilation (NIV) and occasionally invasive mechanical ventilation are required for managing these patients^1–5^. During the major outbreak of SARS, it was found that procedures related to tracheal intubation^6^, oxygen administration ≥6 L/min, and NIV were independent risk factors for super-spreading nosocomial outbreaks affecting many healthcare workers in Hong Kong and Guangzhou, China^7^. In a systematic review of aerosol generating procedures that might increase the risk of nosocomial transmission of SARS to healthcare workers, mask ventilation and oro-tracheal suctioning increased the odds ratios (95% confidence intervals, CI) to 2.8 (1.3–6.4) and 6.2 (2.2–18.1), respectively^8^. In this respect, we have previously shown that the dispersion distances of exhaled plume along the sagittal and transverse planes were 200 and 220 mm, respectively, when bag-mask ventilation was attempted in a human-patient-simulator (HPS)^9^. Although the addition of a viral-bacterial filter eliminated forward leakage of exhaled air from the expiration diverter, leakage at the interface between the mask and the patient’s face was increased to 340 mm along the transverse plane. It is unclear if the increased leakage along the transverse plane was due to the higher resistance with the filter or related to inefficient mask seal^9^. In addition, oro-tracheal suctioning may trigger coughing bouts that could further increase the spread of potentially infectious droplets. However, effective suction may limit the dispersion. Further studies are therefore required to examine if there are technical or human factors involved that would provide useful information on infection control during resuscitation of potentially infected patients with impending respiratory arrest.

The objectives of this study were to evaluate the extent and direction of exhaled air dispersion during attempted bag-mask ventilation by healthcare workers of various experience during simulated resuscitation of the HPS. In addition, we also estimated the spread of exhaled air during episodes of coughing bouts triggered by oro-tracheal suctioning.

## Results

Our research team of investigators, consisting of a respiratory physician, an anesthesiologist/intensivist, and an aerodynamic architect that have jointly conducted and published a series of infection control experiments on a HPS (HPS 6.1, CAE Healthcare, Sarasota, FL) to display exhaled air dispersion quantitatively using a laser visualization technique during application of common respiratory therapies in different hospital ventilation settings^10–16^. In this study, we characterized the pattern of dispersion of potentially infected aerosols during two commonly performed procedures during resuscitation.

### Bag-mask ventilation performed by different groups of healthcare providers

**Figure 1:**
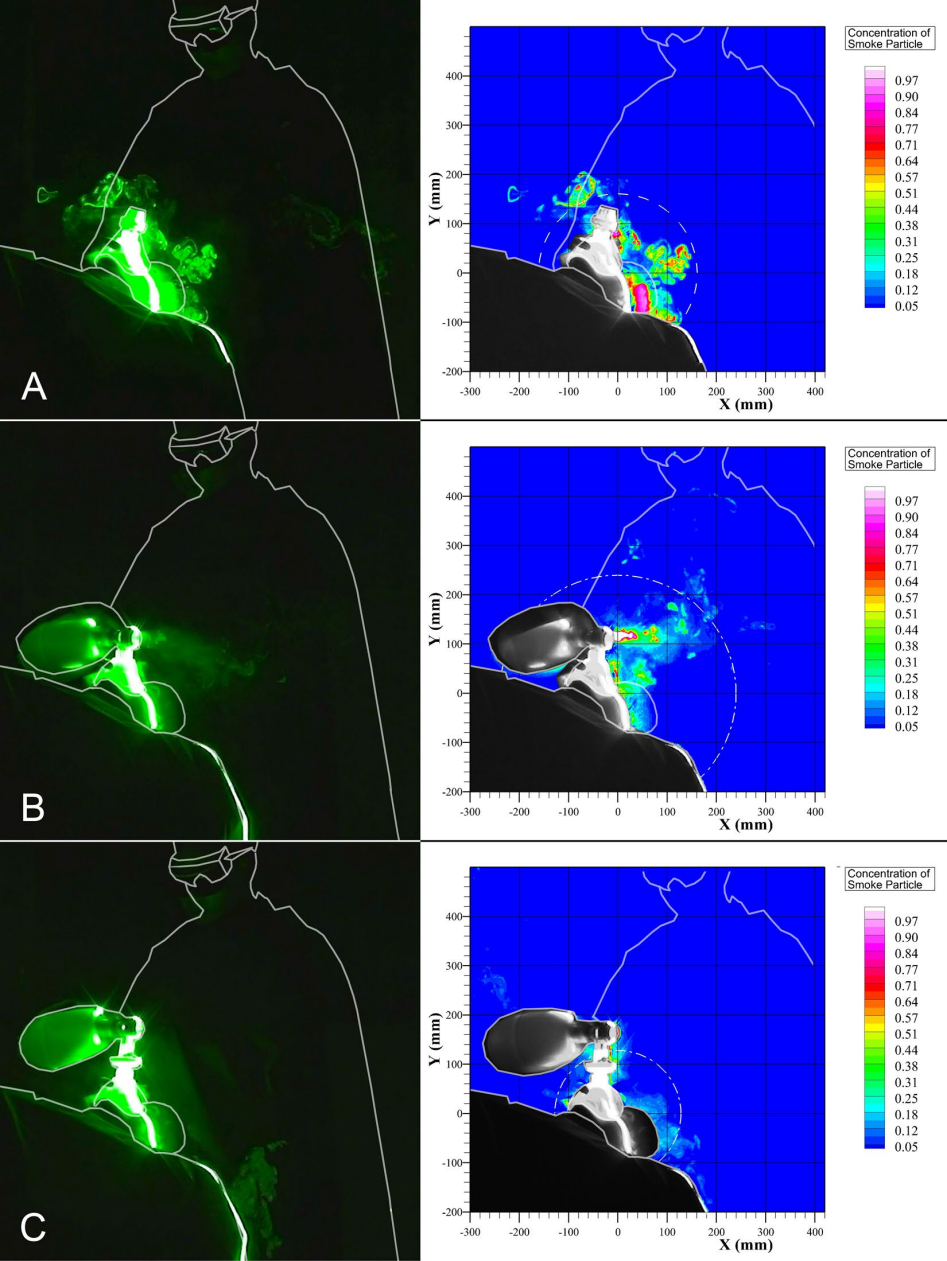
Bag-mask ventilation performed by an anaesthesiologist using (**A**) Laerdal silicone resuscitator (left panel) and the calculated normalized concentration of the smoke particle in the transverse plane of the expiration port (right panel); (**B**) Ambu silicone resuscitator and (**C**) Ambu silicone resuscitator with addition of a breathing filter, respectively.

Figure 1 displays the typical images of exhaled air leakage through the face and mask interface in the transverse plane during bag-mask ventilation. The mean dispersion distances in the transverse plane are summarized in Table 1. In this analysis, nurses produced larger leak than other healthcare workers. The mean difference (95% CI) between nurses and anesthesiologists/intensivists, respiratory physicians or medical students, adjusted for the devices used, were 69 (42–96) mm, 72 (45–99) mm and 67 (40–94) mm, respectively, all *p* < 0.001 (Supplementary Table 1). Dispersion distances were however similar among anesthesiologists/intensivists, respiratory physicians and medical students, *p* = 0.974. Overall, use of Laerdal silicone resuscitator produced less dispersion compared with Ambu silicone resuscitator, mean difference (95% CI) was 44 (21–68) mm, *p* < 0.001. The addition of breathing filter to the Ambu silicone resuscitator reduced leak among anesthesiologists/intensivists, *p* < 0.001, respiratory physicians, *p* = 0.009 and medical students, *p* = 0.011, but not with nurses, *p* > 0.999 (Supplementary Table 2). There was significant interaction between healthcare workers and choice of ventilation devices, *p* = 0.042. Overall, exhaled air leak is the largest with nurses using the Ambu silicone resuscitator with an average dispersion distance of 267 ± 44 mm.

**Table 1:** Dispersion distance of exhaled air during bag-mask ventilation.

Group	No. of providers	Exhaled air dispersion distance (mm)*		
		Laerdal silicone resuscitator	Ambu silicone resuscitator	Ambu silicone resuscitator with addition of breathing filter
Anesthesiologists/Intensivists	5	161 ± 5	242 ± 20	128 ± 21
Respiratory physicians	5	187 ± 17	210 ± 48	148 ± 17
Nurses	5	230 ± 47	267 ± 44	241 ± 62
Medical students	5	175 ± 54	234 ± 51	129 ± 33

Values are mean ± standard deviations.

*Measured at 20% normalized concentration.

### Coughing bouts during oro-tracheal suctioning

**Figure 2:**
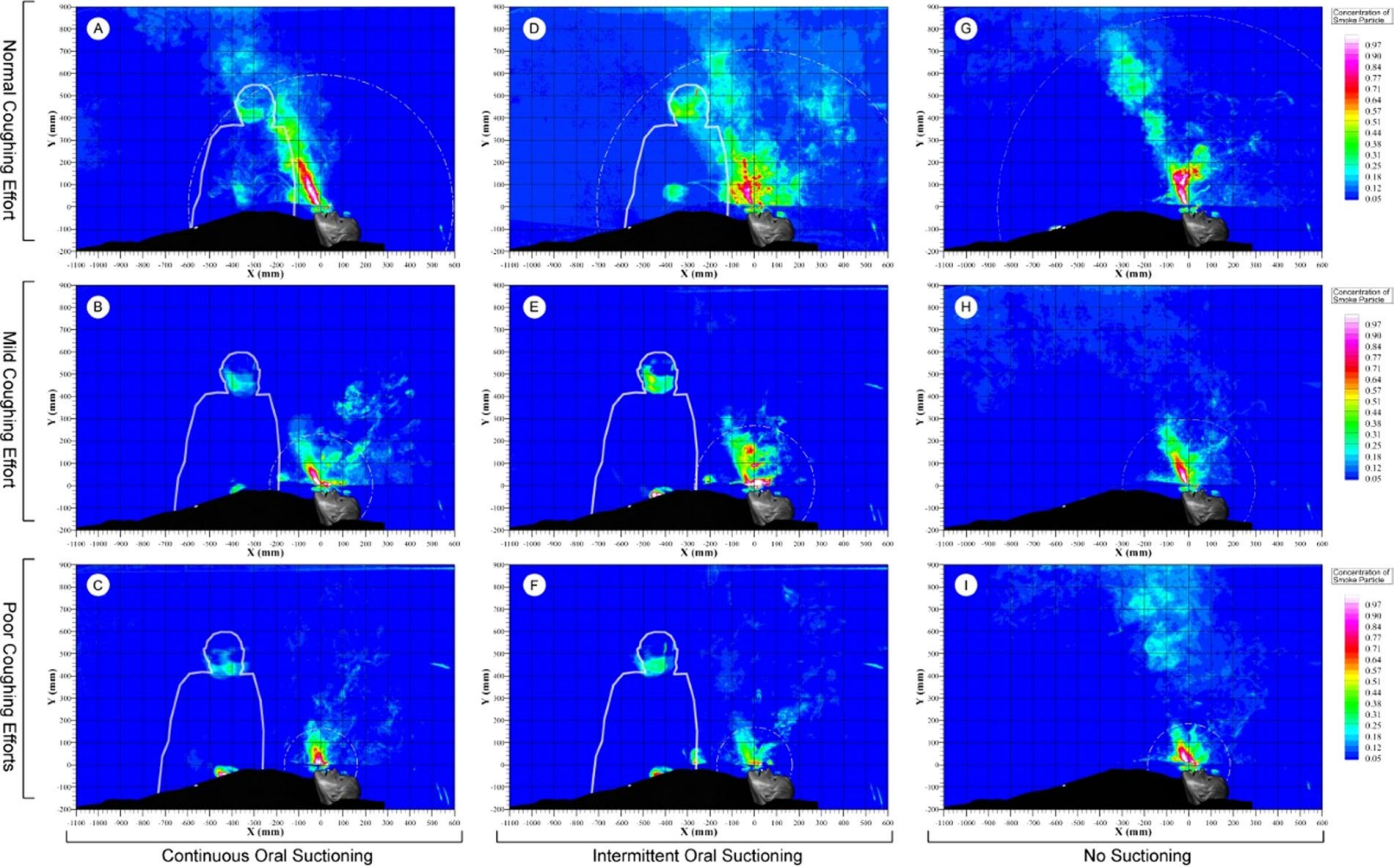
Results of exhaled air dispersion in the sagittal plane during normal, mild and poor coughing efforts generated by short bursts of oxygen flow at 650, 320, and 220 L/min, respectively, in a human patient simulator with continuous (panels A–C), intermittent oral suctioning (panels D–F) and without oral suctioning (panels G–I).

**Figure 3:**
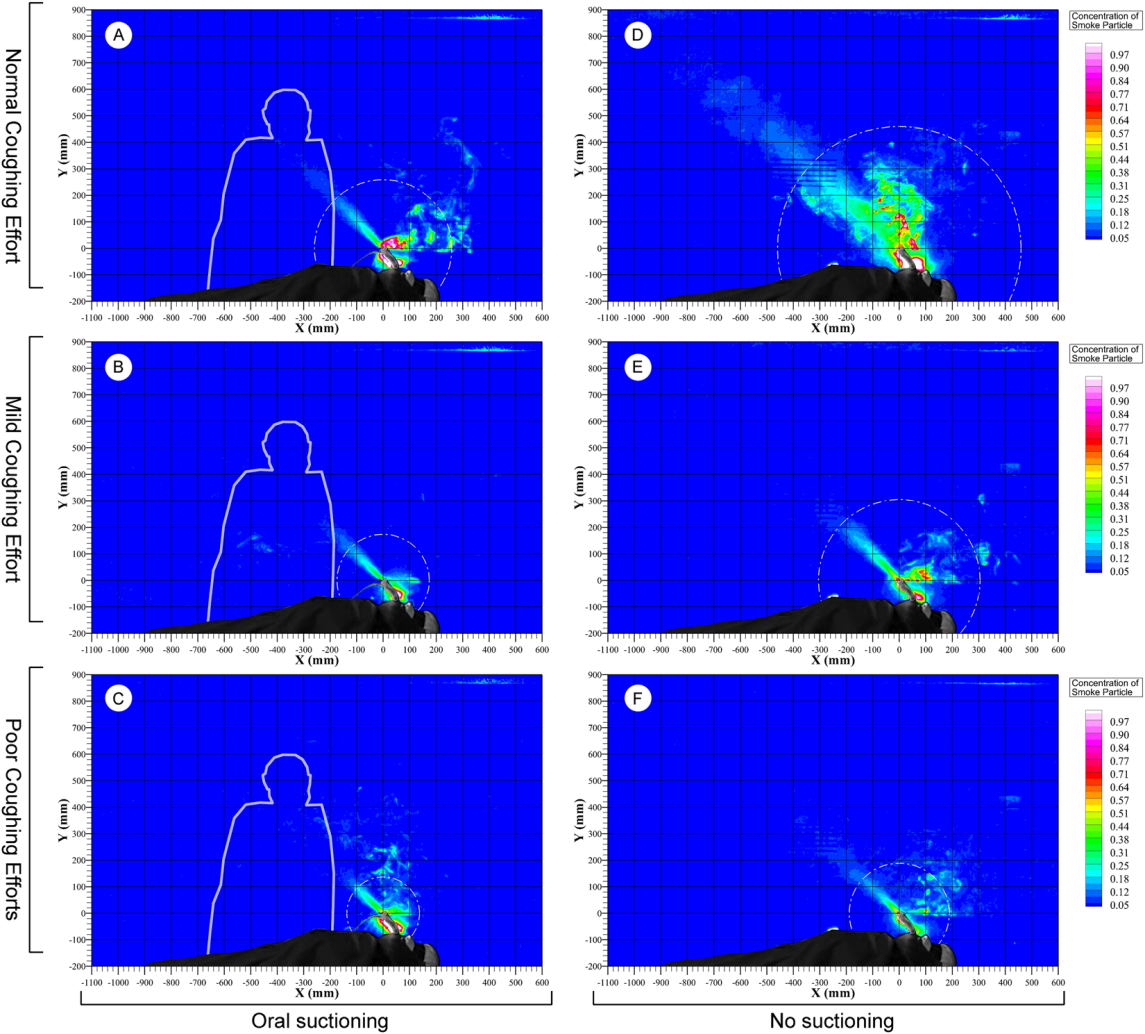
Results of exhaled air dispersion in the sagittal plane during normal, mild and poor coughing efforts generated by short bursts of oxygen flow at 650, 320, and 220 L/min, respectively, in a human patient simulator following tracheal intubation with (panels A–C) and without tracheal suctioning (panels D–F).

**Figure 4:**
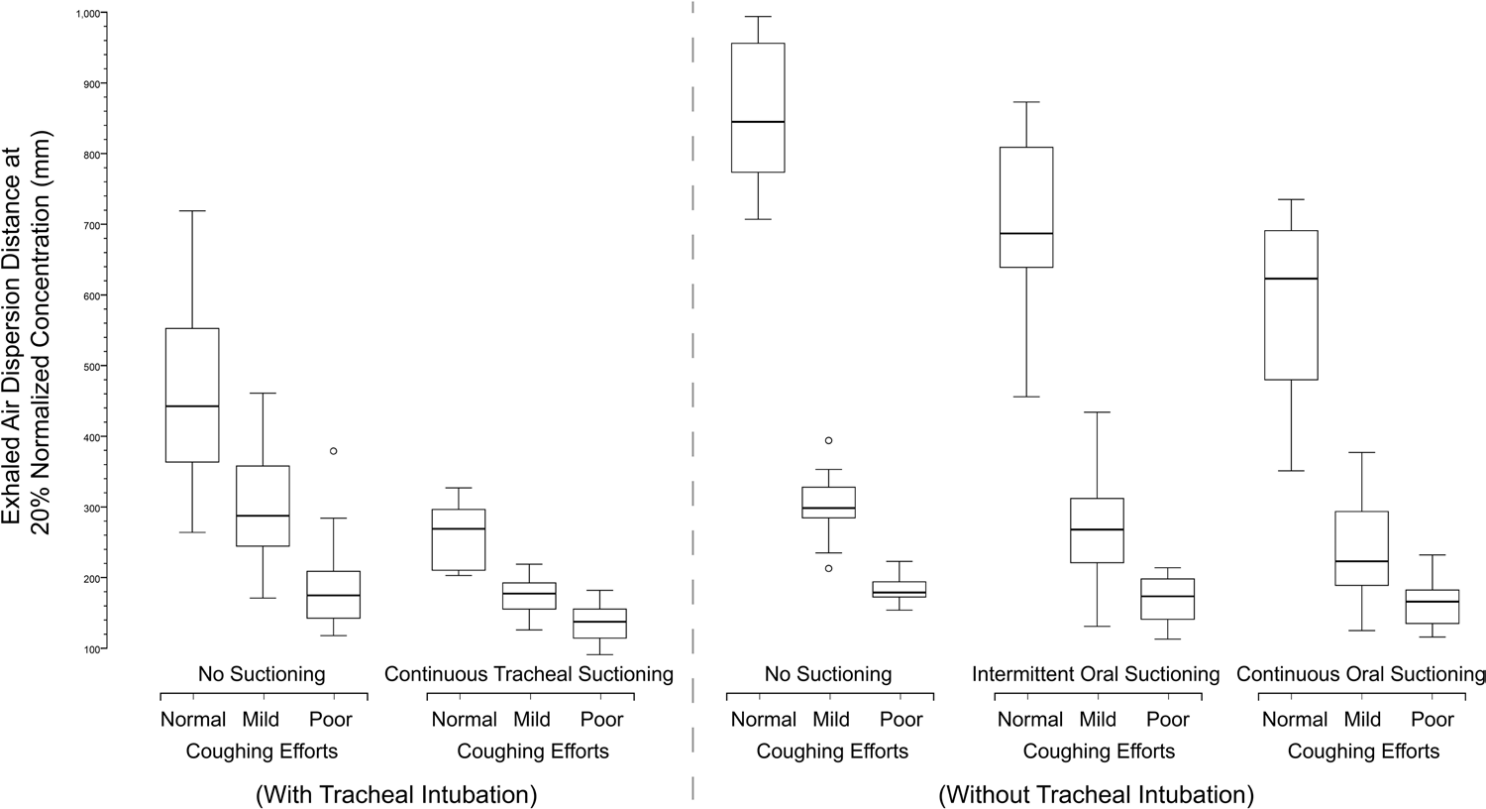
Changes of dispersion distances of exhaled air in the median sagittal plane during normal, mild and poor cough before and after oro-tracheal suction in a human patient simulator with and without tracheal intubation. Box and whiskers plot: the upper and lower edges of the boxes indicate the interquartile ranges, the line through box is the median value, the whiskers are the 5% and 95% centiles and the closed circles are outliers.

We then showed that exhaled air dispersion decreased with worsening coughing efforts (Figs 2 and 3). Before tracheal intubation, exhaled air leaked through the mouth to a distance of 860 ± 93 mm during normal cough. This was reduced to 298 ± 43 mm in mild coughing effort and 185 ± 19 mm with poor coughing effort, *p* < 0.001. Following tracheal intubation, the dispersion distance of exhaled air after a normal cough was 460 ± 127 mm. This was decreased to a distance of 305 ± 77 mm in mild coughing effort and 188 ± 63 mm in poor coughing effort, *p* < 0.001 (Supplementary Table 3). In cases without tracheal intubation, continuous suctioning reduced spread better than intermittent suctioning, adjusted for coughing efforts, *p* < 0.001, (Fig. 4, Supplementary Tables 3 and 4). For example, exhaled air dispersion distance without intubation during normal coughing efforts was 708 ± 105 mm with intermittent suctioning vs 595 ± 122 mm with continuous suctioning (*p* = 0.005). On average, suctioning decreased exhaled air dispersion by >32% (range: 8.2–73.0%, Supplementary Table 4). There was interaction between coughing efforts and suctioning, *p* < 0.001.

## Discussion

In this study, we have examined the dispersion of exhaled air, marked by fine smoke particles, from two commonly performed procedures during resuscitation for respiratory failure, viz bag-mask ventilation and oro-tracheal suctioning. During bag-mask ventilation, our data confirmed the importance of technical skills to minimize exhaled air dispersion. Inexperienced healthcare workers may increase spread of exhaled air by 40%. The medical students who took part in this study had completed a 2-week intensive training course in resuscitation not long before this study and they might be more experienced in performing manual ventilation than the registered nurses who normally worked at pulmonary function laboratory and seldom got involved in active resuscitation. Although the addition of a breathing filter increased the bulk of the device, experienced providers, such as anesthesiologists/intensivists and respiratory physicians, were able to overcome the difficulty to prevent an increase in mask leak. Our data also indicated that coughing during oro-tracheal suctioning could produce substantial dispersion of potentially infected exhaled air. Nevertheless, suctioning reduced the spread of exhaled air during coughing bouts by >32% whereas continuous suctioning could reduce exhaled air distances more effectively than intermittent suctioning.

Among the respiratory procedures, tracheal intubation *per se* or in combination with other procedures (e.g. cardiopulmonary resuscitation or bronchoscopy) was consistently associated with an increased risk of infection transmission through aerosol generation^17^. A study that measured the amount of influenza A(H1N1)2009 RNA in aerosols in the vicinity of H1N1 positive patients undergoing aerosol-generating procedures showed that bronchoscopy and respiratory and airway suctioning were the most definite procedures to produce aerosols above background baseline values^18^. A study of symptomatic adult outpatients has shown that a significant proportion of patients with influenza A could release small airborne particles containing viable virus into the environment through coughing^19^. Another study testing quantitative impaction air samples, which were taken at various distances from patients infected with influenza, by rapid test and polymerase chain reaction has shown that healthcare workers within 1.83 m of patients with influenza could be exposed to infectious doses of influenza virus, primarily in small-particle aerosols (diameter, <4.7 µm) without aerosol-generating procedures whereas influenza virus release was associated with high viral loads in nasopharyngeal samples (shedding), coughing, and sneezing^20^. Our data highlight the importance of continuous suctioning to reduce the spread of aerosols when performing aerosol-generating procedures. In this respect, open airway toileting should be performed with continuous suctioning when closed circuit suctioning is not available on the medical wards for patients with pneumonia of unknown etiology.

There are limitations of our study. Firstly, the use of smoke particles as markers for exhaled air in a HPS model may not reliably reflect trajectory of respiratory droplets. However, a safe marker that can be introduced into human lungs for study is currently not available. Moreover, water content in the respiratory droplets may be evaporated and hence produce small droplet nuclei that are suspended in air. Nevertheless, the smoke particles used in this study mark the continuous air phase, and therefore our data indicated the anticipated “upper limits” of droplet dispersion^10–16,21^.

We measured exhaled air dispersion distance using a threshold of 20% normalized smoke concentration because this is a common procedure in flow visualization studies^22^. The arbitrary threshold allows us to eliminate background contamination and provides a clear and distinctive contour for defining the boundary of dispersion. Using this method, exhaled air was shown to disperse by 0.8 and 1.0 m after application of jet nebulizer and non-invasive ventilation, respectively^11,13,14^. These data are remarkably consistent with the dispersion of 1.0 m, measured by droplet counts, in patients with influenza and chronic lung diseases receiving the same respiratory therapies^23^.

## Conclusions

In summary, continuous suctioning is the most effective method to prevent spread of exhaled air during aerosol generating procedures, including coughing, for management of respiratory failure. Continuous suctioning is therefore recommended to minimize the risk of nosocomial transmission when performing intubation and other aerosol-generating procedures such as bronchoscopy or open sputum suction via tracheostomy in patients with pneumonia and respiratory failure of unknown cause. Mask fit is important in preventing exhaled air leakage during bag-mask ventilation.

## Methods

**Figure 5:**
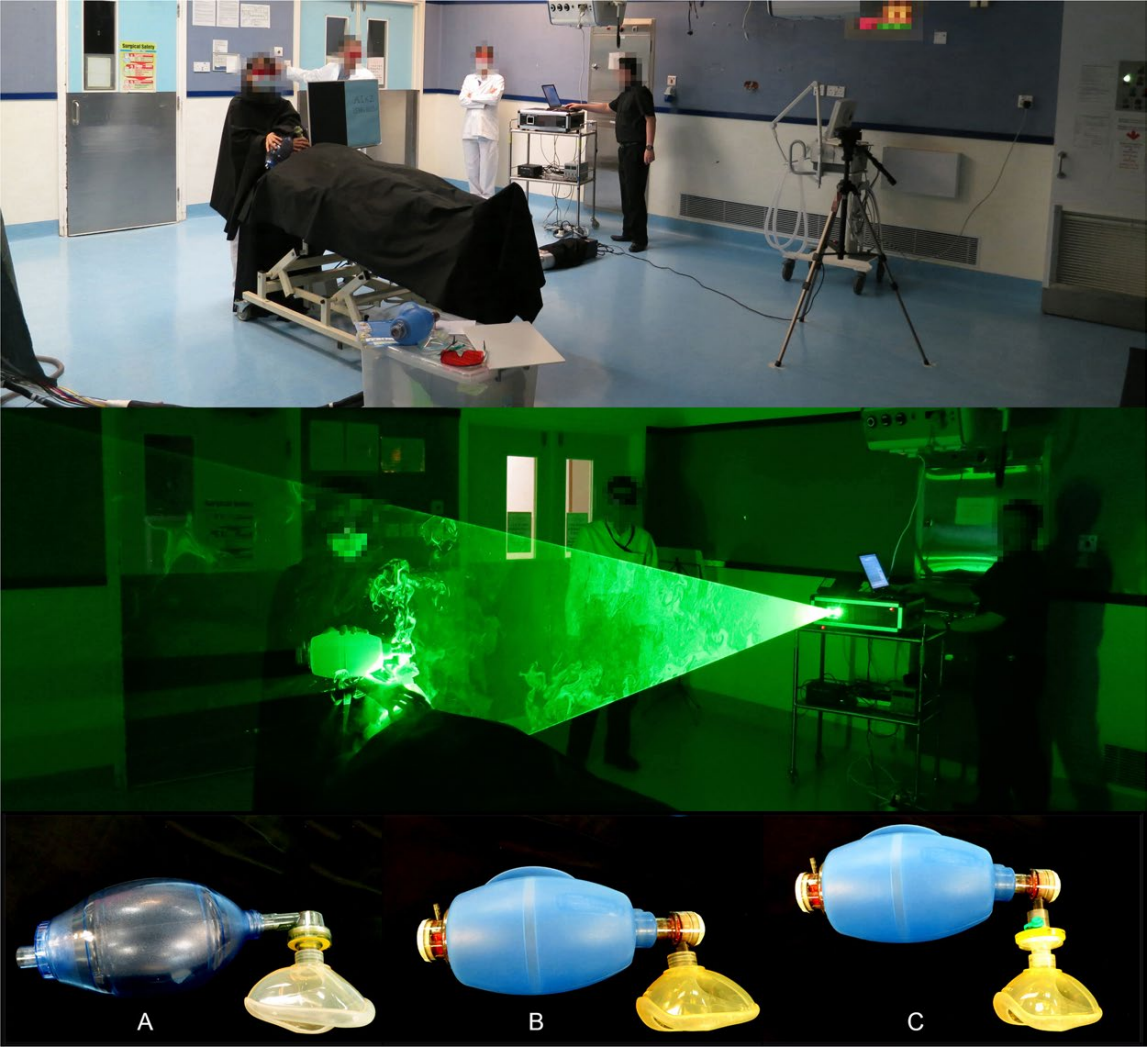
Experimental setup of bag-mask ventilation (top panel). Exhaled air dispersion highlighted by the laser light sheet during bag-mask ventilation (lower panel) with (**A**) Laerdal silicone resuscitator, (**B**) Ambu silicone resuscitator and (**C**) Ambu silicone resuscitator with the addition of a breathing filter on the human patient simulator.

In the first experiment, we investigated bag-mask ventilation during resuscitation with the HPS lying supine to simulate in-hospital resuscitation for respiratory failure. In order to evaluate human factors involved in the exhaled air dispersion during bag-mask ventilation, four groups of healthcare workers – anesthesiologists/intensivists (*n* = 5), respiratory physicians (*n* = 5), registered nurses who worked at the pulmonary function laboratory (*n* = 5) and medical students (*n* = 5) took part in the experiments as providers for resuscitation. All providers were asked to ventilate the HPS using three devices in random sequence – (1) a Laerdal silicone resuscitator (Laerdal Medical, Wappingers Falls, NY); (2) an Ambu oval silicone resuscitator (Ambu A/S, Ballerup, Demark) with and (3) without addition of a breathing filter (Inter-Guard breathing filter, Intersurgical, Berkshire, UK). A size 4 open cuff silicone facemask (Ambu A/S, Ballerup, Demark) was used in all scenarios. All providers were allowed to practice bag-mask ventilation on the HPS using the three devices for a maximum duration of 30 minutes before conducting the experiment. For each device, the provider was instructed to deliver 10–12 ventilations/min for 3 minutes. All providers wore protective eye goggles while performing bag-mask ventilation. Figure 5 shows the room set-up for the experiment.

**Figure 6:**
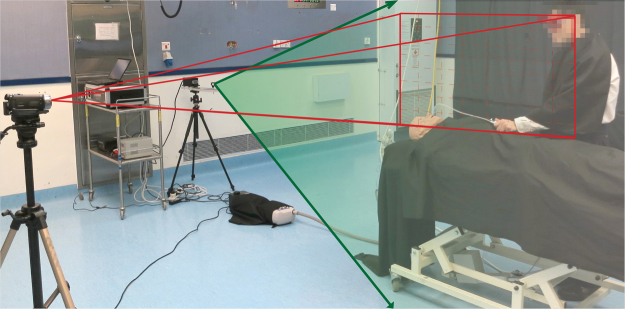
Experimental setup to evaluate coughing through a tracheal tube during tracheal suctioning. The human patient simulator was positioned in the supine position. Exhaled air plume was indicated with intrapulmonary smoke and was revealed by laser light sheet in the sagittal plane (shown with the green section/arrows). Images were captured by high-definition camera positioned in a perpendicular position (red area) for data analysis.

In the second experiment, we estimated the dispersion of exhaled air from coughing during airway toileting. We inserted a size 10 FG suction catheter into the oral cavity. In order to simulate normal, mild and poor coughs, we generated short bursts (20 ms) of airflow through the catheter at 650, 320, and 220 L/min, respectively^21^. The trachea was then intubated with a 9 mm tracheal tube (Fig. 6). Experiments were then repeated with coughing through the tracheal tube. Changes of spread of exhaled air were investigated with suction at −45 kPa continuously and then intermittently.

### Flow visualization

Smoke particles of <1 μm in diameter, produced by a M-6000 smoke generator (N19, DS Electronics, Sydney, Australia) was used to highlight exhaled airflow as previously described^10–16,21,24^. A 6 FG catheter was inserted into the right main bronchus of the HPS to deliver smoke particles. After mixing with alveolar gas, smoke particles were exhaled through the normal airway passage. Leakage jet plume were then illuminated by a green (532 nm wavelength) laser light-sheet using a continuous pulse, diode-pumped solid state laser generator (OEM UGH-800 mW, Lambdapro Technologies, China)^10–16,21^. Assuming the intensity of the laser and the size or shape of the smoke particles remained constant during examination^24^, the intensity of light scattered by the smoke particles indicated the exhaled air smoke concentration. The laser light-sheet was programmed to scan through the sagittal and transverse planes of the HPS to evaluate leakage plume above and lateral to the mask of the HPS^10–16,21^. In this study, we reported the plane of vision that contained the largest dispersion distance.

Images of leakage jet plume are captured by high definition video camera (1,440 × 1,080 pixels per frame). We analyzed the image frames from the beginning of coughing or inspiration during each ventilation. Intensity analysis was performed on individual frame stored in gray scale bitmap format. In this analysis, we subtracted the background intensity with images taken with the laser turned off. Light intensity was then averaged over all frames. The resulting image was the total scattered light intensity and was proportional to the smoke particle concentration. This was then normalized against the spot with the highest intensity in the exhaled jet plume to produce a contour map showing normalized particle concentration^10–16,21^. Given that the smoke particles originated from the lungs and airway, the concentration contour indicated the probability of encountering potentially infected exhaled air that came from within the mask or patient’s respiratory system. In this respect, a 0 value indicated a region with no measurable smoke particle or exhaled air^10–16,21^.

### Statistical analysis

The dispersion distance was expressed as mean ± standard deviation. A general linear model was used to compare the difference in exhaled air dispersion among different mask ventilation systems (Laerdal and Ambu resuscitator with or without breathing filter) and that performed by different groups of healthcare workers (anesthesiologists/intensivists, respiratory physicians, nurses, or medical students).

Similar model was used to compare the differences in exhaled air dispersion after coughing with or without tracheal intubation and the effect of continuous and intermittent orotracheal suctioning during different degree of lung injury (mild, moderate *vs* severe). A *p* value <0.05 is considered as statistically significant. The study received non-ionizing radiation safety approval by the Chinese University of Hong Kong (N/DSCH/RFCID 2012).

### Data availability

The datasets used and/or analyzed during the current study are available from the corresponding author on reasonable request.

## Electronic supplementary material

Supplementary Tables
