# Supplementary material for: Exhaled air dispersion during bag-mask ventilation and sputum suctioning - Implications for infection control
Source: Sci Rep. 2018 Jan 9;8:198. doi: 10.1038/s41598-017-18614-1 (PMC5760517; doi:10.1038/s41598-017-18614-1)
Supplement: Supplementary file 1 — Supplementary Tables [file 41598_2017_18614_MOESM1_ESM.pdf]

Supplementary Tables 1-4

Exhaled air dispersion during bag-mask ventilation and sputum suctioning - Implications for infection control

Matthew TV Chan, Benny K Chow, Thomas Lo, Fanny W Ko, Susanna S Ng, Tony Gin, David S Hui

Supplementary Table 1. Differences of dispersion distance of exhaled air during bag-mask ventilation between nurses and anesthesiologists/intensivists, respiratory physicians or medical students, using Laerdal silicone resuscitator; Ambu silicone resuscitator with and without breathing filter.

	Mean difference (95%CI) (mm)	**p* value
**Overall**		
Nurses – Anesthesiologists/Intensivists	69 (42 – 96)	<0.001
Nurses – Respiratory physicians	72 (45 – 99)	<0.001
Nurses – Medical students	67 (40 – 94)	<0.001
**Laerdal silicone resuscitator**		
Nurses – Anesthesiologists/Intensivists	70 (-0.5 – 140)	0.052
Nurses – Respiratory physicians	44 (-26 – 114)	0.476
Nurses – Medical students	23 (-15 – 125)	0.187
**Ambu silicone resuscitator**		
Nurses – Anesthesiologists/Intensivists	25 (-43 – 94)	>0.999
Nurses – Respiratory physicians	80 (12 – 149)	0.017
Nurses – Medical students	33 (-36 – 101)	>0.999
**Ambu silicone resuscitator with breathing filter**		
Nurses – Anesthesiologists/Intensivists	114 (42 – 185)	0.001
Nurses – Respiratory physicians	93 (22 – 165)	0.007
Nurses – Medical students	112 (41 – 184)	0.001

*General linear model with *post hoc* Bonferroni test.
CI = confidence intervals

Supplementary Table 2. Differences of dispersion distance of exhaled air during bag-mask ventilation using Laerdal silicone resuscitator, stratified by anesthesiologists/intensivists, respiratory physicians, medical students and nurses.

	Mean difference (95%CI) (mm)	**p* value
**Overall**		
Ambu silicone resuscitator – Laerdal silicone resuscitator	44 (21 – 68)	<0.001
Ambu silicone resuscitator – Ambu silicone resuscitator with filter	71 (42 – 100)	<0.001
Laerdal silicone resuscitator – Ambu silicone resuscitator with filter	27 (-2 – 56)	0.074
**Anesthesiologists/Intensivists**		
Ambu silicone resuscitator - Laerdal silicone resuscitator	81 (51– 111)	<0.001
Ambu silicone resuscitator - Ambu silicone resuscitator with filter	114 (84 – 144)	<0.001
Laerdal silicone resuscitator – Ambu silicone resuscitator with filter	33 (3 – 63)	0.029
**Respiratory physicians**		
Ambu silicone resuscitator - Laerdal silicone resuscitator	0 (-29 – 29)	>0.999
Ambu silicone resuscitator - Ambu silicone resuscitator with filter	39 (10 – 69)	0.009
Laerdal silicone resuscitator – Ambu silicone resuscitator with filter	39 (10 – 68)	0.009
**Medical Students**		
Ambu silicone resuscitator - Laerdal silicone resuscitator	59 (-23 – 141)	0.205
Ambu silicone resuscitator - Ambu silicone resuscitator with filter	106 (24 – 188)	0.011
Laerdal silicone resuscitator – Ambu silicone resuscitator with filter	47 (-35 – 129)	0.419
**Nurses**		
Ambu silicone resuscitator - Laerdal silicone resuscitator	37 (-54 – 128)	0.858
Ambu silicone resuscitator - Ambu silicone resuscitator with filter	26 (-65 – 117)	>0.999
Laerdal silicone resuscitator – Ambu silicone resuscitator with filter	-11 (-102 – 80)	>0.999

*General linear model with *post hoc* Bonferroni test.
CI = confidence intervals

Supplementary Table 3. Dispersion distances of exhaled air in the median sagittal plane during normal, mild and poor cough before and after oro-tracheal suctioning in a human patient simulator with and without tracheal intubation

	Coughing efforts		
	Normal	Mild	Poor
Without tracheal intubation			
No suctioning	860 ± 93	290 ± 43	185 ± 19
	845 (771 – 959)	299 (284 – 328)	179 (172 – 195)
Intermittent suctioning	708 ± 105	269 ± 71	170 ± 33
	687 (638 – 810)	268 (220 – 314)	174 (141 – 199)
Continuous suctioning	595 ± 122	232 ± 70	164 ± 31
	623 (478 – 694)	223 (189 – 295)	166 (134 – 185)
With tracheal intubation			
No suctioning	460 ± 127	305 ± 77	189 ± 63
	443 (363 – 567)	288 (244 – 365)	175 (142 – 212)
Continuous suctioning	259 ± 45	174 ± 26	137 ± 27
	269 (210 – 299)	178 (155 – 193)	138 (113 – 156)

Values are mean ± standard deviations, median (interquartile range).

Supplementary Table 4. Effect of Continuous or intermittent suctioning on the dispersion distance of exhaled air with and without tracheal intubation in the sagittal plane.

	Mean difference (95%CI) mm	Percentage change in dispersion distance	**p* value
**Tracheal intubation**			
Overall			
No suctioning – continuous suctioning	128 (103 – 153)	-40.3%	<0.001
Normal coughing effort			
No suctioning – continuous suctioning	201 (140 – 262)	-43.7%	<0.001
Mild coughing effort			
No suctioning – continuous suctioning	305 (278 – 331)	-43.0%	<0.001
Poor coughing effort			
No suctioning – continuous suctioning	52 (21 – 83)	-27.6%	0.002
**Without tracheal intubation**			
Overall			
No suctioning – continuous suctioning	305 (270 – 340)	-48.0%	<0.001
No suctioning – intermittent suctioning	253 (218 – 288)	-39.8%	<0.001
Intermittent – continuous suctioning	52 (17 – 87)	-13.6%	0.001
Normal coughing effort			
No suctioning – continuous suctioning	266 (182 – 349)	-30.9%	<0.001
No suctioning – intermittent suctioning	152 (68 – 236)	-17.7%	<0.001
Intermittent – continuous suctioning	113 (29 – 197)	-13.6%	0.005
Mild coughing effort			
No suctioning – continuous suctioning	628 (567 – 690)	-73.0%	<0.001
No suctioning – intermittent suctioning	591 (530 – 653)	-68.8%	<0.001
Intermittent – continuous suctioning	37 (-25 – 98)	-13.6%	0.441
Poor coughing effort			
No suctioning – continuous suctioning	21 (-1 – 43)	-11.2%	0.065
No suctioning – intermittent suctioning	15 (-7 – 37)	-8.2%	0.297
Intermittent – continuous suctioning	6 (-16 – 28)	-3.6%	>0.999

*General linear model with *post hoc* Bonferroni test.
CI = confidence intervals
